# Botulinum Neurotoxin Type A Induces TLR2-Mediated Inflammatory Responses in Macrophages

**DOI:** 10.1371/journal.pone.0120840

**Published:** 2015-04-08

**Authors:** Yun Jeong Kim, Jeong-Hee Kim, Kwang-Jun Lee, Myung-Min Choi, Yeon Hee Kim, Gi-eun Rhie, Cheon-Kwon Yoo, Kiweon Cha, Na-Ri Shin

**Affiliations:** Division of High-risk Pathogen Research, Center for Infectious Diseases, Korea National Institute of Health, Korea Centers for Disease Control and Prevention, Cheongju, Korea; University of Wisconsin, Food Research Institute, UNITED STATES

## Abstract

Botulinum neurotoxin type A (BoNT/A) is the most potent protein toxin and causes fatal flaccid muscle paralysis by blocking neurotransmission. Application of BoNT/A has been extended to the fields of therapeutics and biodefense. Nevertheless, the global response of host immune cells to authentic BoNT/A has not been reported. Employing microarray analysis, we performed global transcriptional profiling of RAW264.7 cells, a murine alveolar macrophage cell line. We identified 70 genes that were modulated following 1 nM BoNT/A treatment. The altered genes were mainly involved in signal transduction, immunity and defense, protein metabolism and modification, neuronal activities, intracellular protein trafficking, and muscle contraction. Microarray data were validated with real-time RT-PCR for seven selected genes including *tlr2*, *tnf*, *inos*, *ccl4*, *slpi*, *stx11*, and *irg1*. Proinflammatory mediators such as nitric oxide (NO) and tumor necrosis factor alpha (TNFα) were induced in a dose-dependent manner in BoNT/A-stimulated RAW264.7 cells. Increased expression of these factors was inhibited by monoclonal anti-Toll-like receptor 2 (TLR2) and inhibitors specific to intracellular proteins such as c-Jun N-terminal kinase (JNK), extracellular signal–regulated kinase (ERK), and p38 mitogen–activated protein kinase (MAPK). BoNT/A also suppressed lipopolysaccharide-induced NO and TNFα production from RAW264.7 macrophages at the transcription level by blocking activation of JNK, ERK, and p38 MAPK. As confirmed by TLR2-/- knock out experiments, these results suggest that BoNT/A induces global gene expression changes in host immune cells and that host responses to BoNT/A proceed through a TLR2-dependent pathway, which is modulated by JNK, ERK, and p38 MAPK.

## Introduction

Botulinum neurotoxin (BoNT), produced by *Clostridium botulinum*, is widely recognized as the most poisonous substance in nature. Seven immunologically distinct serotypes of the toxin have been identified and designated A through G [1]. Among them, toxin types A, B, E, and more rarely, F, are known to be responsible for natural botulism in humans. Humans are usually exposed to these neurotoxins through food poisoning, however there are some incidences of infant botulism from intestinal colonization as well as wound botulism [2]. More recently, the threat of botulinum toxin used as a biological weapon [3] that would cause inhalational botulism has also been identified [4]. Botulinum toxin is a dichain polypeptide that consists of a 100-kDa heavy chain joined by a single disulfide bond to a 50-kDa light chain [5]. While heavy chain of the toxin plays dual roles of receptor binding and translocation, the light chain of the toxin is a zinc endopeptidase that blocks acetylcholine-containing vesicles from fusing with the presynaptic terminal membrane of the motor neuron. The actions of the light chain and their effects on presynaptic vesicles result in flaccid muscle paralysis [6].

Macrophages are critical components of the immune system and play significant roles in both the innate and acquired immune responses [7]. These cells constitute the first line of defense, with the ability to detect and recognize potential pathogens and become activated to produce proinflammatory cytokines and antimicrobial small molecules such as NO and defensins [8–10]. Microbial recognition by macrophages is mediated by pattern-recognition receptors such as the Toll-like receptors (TLRs), which bind molecules with repetitive patterns in microorganisms [11,12]. TLRs link microbial recognition to the activation of antigen-presenting cells, the specialized cells involved in T-lymphocyte activation [7,13]. TLRs are also involved in initiation of adaptive immunity through a signaling pathway that culminates in the activation of nuclear factor-κB (NF-κB) transcription factors and mitogen-activated protein kinases (MAPKs) including c-Jun N-terminal kinase (JNK), p38, and extracellular signal-regulated kinase (ERK) [12,14]. These proteins ultimately regulate genes that are involved in the activation of microbial killing mechanisms, apoptosis, antigen recognition, pro- and anti-inflammatory cytokines, and chemokines [15].

In mammals, the TLR family consists of 13 members (TLR1–13), and several ligands recognized by them have been identified [16]. For instance, TLR4 mediates lipopolysaccharide (LPS)-induced signal transduction, whereas TLR2 responds to lipoproteins and peptidoglycans from gram-positive bacteria [17–19]. More recently, several factors secreted from microbes have been shown to be dependent on TLR2 for induction of immune effects. These include group B streptococcal soluble factors [20], *Bacillus anthracis* protective antigen [21], and the pentameric B subunit of the *Escherichia coli* LT-IIb enterotoxin [22]. When a human is exposed to BoNT, the toxin is absorbed into the circulation from a mucosal surface, and then it directly and rapidly targets the presynaptic terminal before the host immune system is evoked. Furthermore, BoNT has been described as inducing little inflammation [23]. These characteristics remain a substantial obstacle to studies on the *in vivo* inflammatory effects of the active toxin on the host. Likewise, few reports have been published on the *in vitro* effects of botulinum toxin on host immune cells. Several previous studies have documented cell-specific responses to BoNT.

Therefore, the aim of this study was to examine global host responses following the interaction between BoNT/A and host immune cells. The murine alveolar macrophage cell line, RAW264.7, was used in this study because aerosolized botulinum toxin would encounter alveolar macrophages in the lung. Aerosolized botulinum toxin can be absorbed through the lungs of monkeys, and this may occur in the case of a terrorist attack [24]. In the present study, we used microarray technology to define the global transcript profile of macrophages exposed to BoNT/A to provide information about host defense mechanisms and the early host response to BoNT/A. We also characterized the effects of BoNT/A on LPS-stimulated macrophages. Our data indicate that BoNT/A suppresses LPS-induced inflammatory responses in RAW264.7 cells and that the macrophage response to BoNT/A stimulation proceeds through TLR2-dependent pathways, which are modulated by JNK, ERK, and p38. Together, our findings provide significant new insight into the early molecular events in the host response upon exposure to BoNT/A and advance the understanding of the molecular basis of innate immune cell activation after BoNT/A exposure.

## Materials and Methods

### Animals

Female TLR2 -/- knock out mice and control C57BL/6 mice were maintained under a pathogen-free Central Animal Facility of the KNIH. This study was carried out in strict accordance with the recommendations in the Guidelines for the Care and Use of Laboratory Animals of the National institutes of Health. All animal experiments were approved by the KNIH Ethics Committee on the Use and Care of Animals. Bone marrow was isolated after carbon dioxide euthanasia and all efforts were made to minimize suffering.

### BoNT/A Preparation

BoNT/A (1.0 × 10^7^ mouse i.p. LD_50_/mg) was purified from *C*. *botulinum* ATCC19397 [25], and the bioactivity was determined in mice [26]. BoNT/A was further purified upon superdex200 FPLC (Figure A (A) in S1 File). Haemagglutinin-free toxin was obtained from p-amino glucopyranoside-agarose affinity choromatography (Figure A (B) in S1 File). Protein bands were identified by peptide mass finger printing (Figure A (C) and (D) in S1 File).

### Cell culture and treatments

The murine alveolar monocyte/macrophage cell line RAW264.7 (ATCC, Manassas, VA) was grown in complete Dulbecco’s modified Eagle minimal essential medium (DMEM) (Gibco, Gaithersburg, MD) supplemented with 10% fetal bovine serum (Gibco), 2 mM l-glutamine (Gibco), penicillin (100 units/ml), and streptomycin (0.1 mg/ml) to 90% confluence in 75-cm^2^ cell culture flasks (Nunc, Roskilde, Denmark). Cultures were maintained at 37°C in a 5% CO_2_ humidified atmosphere.

### Mouse Bone Marrow-derived Macrophages (BMDMs) Isolation

Cells from the bone marrow of C57BL6 mice were cultured in DMEMs medium (10% FCS) supplemented with 15% MEF conditioned media for 7 days to allow differentiation to macrophages. Conditioned medium was collected from MEF cells incubated in DMEM for 24h, and filtered through a 0.2 μm filter. Conditioned medium samples were added to BMDMs for 24h, after which TNFα and IL-6 expressions were assayed.

### Cytotoxicity detection assay

Cellular cytotoxicity was measured in the different assays using the lactate dehydrogenase CytoTox 96 nonradioactive cytotoxicity assay (Promega, Madison, WI) as described by the manufacturer. Untreated cells were used as a negative control, and completely lysed cells treated with 2% Triton X-100 represented 100% cytotoxicity (positive control). Optical densities were measured at 490 nm with a microplate reader (Tecan, Oberdiessbach, Switzerland) and used to calculate the percentage of cytotoxicity.

### RAW264.7 cell stimulation and total RNA extraction for microarray

RAW264.7 cells (5.0 × 10^5^ cells/ml) were plated in 21.5-cm^2^ dishes (Nunc) in supplemented DMEM. The next day, the medium was replaced with fresh medium containing BoNT/A (1 or 5 nM). After incubation for 0, 2, 4, 6, 8, or 10 h, cells were collected for RNA isolation. Total RNA was extracted from BoNT/A-treated cells using TRIzol reagent (Invitrogen, Carlsbad, CA) and purified using RNeasy columns (Qiagen, Valencia, CA) according to the manufacturers’ protocols. After contaminating DNA was degraded with 20 U RNase-free DNase (Promega), the concentration and purity of RNA samples were evaluated with denaturing gel electrophoresis and by measuring the absorbance at 260 and 280 nm with an Agilent 2100 analyzer (Agilent Technologies, Palo Alto, CA). RNA preparations for microarray were independently performed in triplicate under the same conditions.

### Microarray hybridization and analysis

RNA amplification and the biotin labeling step were performed using the Illumina TotalPrep RNA Amplification kit (Ambion, Austin, TX) according to the manufacturer’s instructions. Briefly, 550 ng total RNA was reverse transcribed to first-strand cDNA with an oligo (dT) primer bearing a T7 promoter and ArrayScript reverse transcriptase. Then, second-strand cDNA was synthesized with DNA polymerase and cleaned up for in vitro transcription. From the double-stranded cDNA templates, biotinylated, antisense-amplified RNA copies of each mRNA (cRNA) were generated with T7 RNA polymerase and a biotin-NTP mix. After purification, the cRNA was quantified using an ND-1000 Spectrophotometer (NanoDrop, Wilmington, DE), and then the labeled cRNA samples (750 ng) were hybridized to Mouse-6 Expression BeadChips (Illumina, San Diego, CA) for 16–18 h at 58°C. The hybridized chips were washed and stained with Amersham fluorolink streptavidin-Cy3 (GE Healthcare Bio-Sciences, Little Chalfont, UK), and the fluorescent images were visualized with an Illumina BeadArray Reader. The array data were extracted using Illumina BeadStudio software (v. 2.1.12) and filtered with a detection *p*-value <0.05. The selected gene signal value was transformed by logarithm and normalized with the quantile method. Genes differentially expressed between BoNT/A-stimulated (2, 4, 6, 8, and 10 h) and control (0 h) samples were identified with both a Local-Pool-Error (LPE) test *p*-value <0.05 (http://bioinformatics.oxfordjournals.org/cgi/reprint/19/15/1945) and a fold change greater than 2.0 in all three independent experiments. The LPE test’s *p*-value was estimated with the LPE Bioconductor package (www.bioconductor.org). Computational hierarchical and *k*-means clustering with complete linkage and Euclidean distance were performed using ArrayAssist (Stratagene, La Jolla, CA). Biological processes were analyzed using the Panther database (http://www.pantherdb.org), and gene ontology and the related signaling pathways were analyzed with KEGG pathway analysis using the David database (http://david.abcc.ncifcrf.gov) and pathwayArchitect software (Stratagene), respectively. Microarray data has been deposited to NCBI GEO public database (Accession number: GSE64390).

### Quantitative reverse transcriptase-polymerase chain reaction (qRT-PCR)

qRT-PCR was used to measure gene expression of *tlr2*, *tnf*, *inos*, *ccl4*, *slp1*, *stx11*, *and irg1* using specific primers and probes (Table 1). Reverse transcription using oligo (dT)_20_ and 500 ng total RNA was performed to generate cDNAs using the Superscript II RT-PCR system (Invitrogen, Karlsruhe, Germany) in accordance with the manufacturer’s instructions. Real-time PCR was performed in 384-well microtiter plates on an ABI Prism 7900HT sequence detection system. The two-step amplification was performed in a 10-μl reaction volume containing 90 nM each primer, 250 nM fluorescence-labeled TaqMan probe, 2 μl three-fold diluted cDNA, and TaqMan Universal PCR Master Mix (Applied Biosystems, Foster City, CA). Samples were run in triplicate, and the data were analyzed with Sequence Detector software (Applied Biosystems). The experimental data were represented as fold changes of gene expression of BoNT/A-stimulated cells (2, 4, 6, 8, and 10 h) relative to control (0 h) samples. mRNA levels for the samples were normalized to GAPDH mRNA levels.

**Table 1:** Genes, specific primers, and probes used for qRT-PCR.

Gene name	Symbol	GenBank accession no.	Gene Expression Assay ID
Toll-like receptor 2	*tlr2*	NM_011905.2	Mm00442346_m1
Tumor necrosis factor	*tnf*	NM_013693	Mm99999068_m1
Nitric oxide synthase 2, inducible	*inos*	NM_010927.1	Mm00440502_m1
Chemokine (C-C motif) ligand 4	*ccl4*	NM_013652	Mm00443111_m1
Secretory leukocyte protease inhibitor	*slpi*	NM_011414.1	Mm00441527_m1
Syntaxin 11	*stx11*	XM_203312.2	Mm01192496_m1
Immunoresponsive gene 1	*irg1*	XM_127883	Mm01224532_m1

### Preparation of BMDM / RAW264.7 cells and supernatants

To examine the stimulatory effect of different concentrations of BoNT/A, BMDM / RAW264.7 macrophages were cultured with 0, 1, 2, 5, and 10 μg/ml BoNT/A. The supernatants were collected and stored at −70°C until assayed for NO and cytokines. We also investigated the effect of BoNT/A or formalin-inactivated BoNT/A (BoNToxoid/A) on LPS-induced RAW264.7 macrophages. BoNToxoid/A was prepared by treating with 0.4% formalin for 5 days at 37°C. Then, 10 μg BoNToxoid/A was administered (i.p.) to four mice, and they were observed for 4 days to confirm that the formalized toxin was completely nontoxic by showing no death or specific symptoms such as muscle spasms, stiffening, or any other abnormal signs during the observation period. RAW264.7 cells (5.0 × 10^5^ cells/ml) were stimulated with 1 μg/ml BoNT/A, 1 μg/ml BoNToxoid/A, or 1 μg/ml LPS (Sigma-Aldrich, St. Louis, MO) for 24 h, and then BoNT/A- or BoNToxoid/A-pretreated cells were incubated with 1 μg/ml LPS for 24 h. The cells and the culture supernatants were taken for qRT-PCR and for TNFα and nitrite analyses. Non-treated cells and fresh medium were used as negative controls. All experiments were performed in triplicate and were independently carried out three times.

### Cytokine analysis

After incubation, the culture supernatants were collected and stored at −70°C until the assay. The levels of TNFα, interleukin (IL)-1β, IL-6, and IL-12 in cell-free supernatants were quantitatively determined using commercial colorimetric sandwich ELISA kits (Pierce, Rockford, IL) according to the manufacturer’s protocol. Data are the means ± standard deviation of the results of three experiments with duplicate samples.

### Nitrite analysis

The production of NO was determined by measuring nitrite accumulation in the culture medium with the Griess reaction. Briefly, 50-μl aliquots of culture supernatants were incubated with equal volumes of Griess reagent, containing 1% sulfanilamide (Sigma) and 0.1% naphthyl ethylenediamine dihydrochloride (Sigma) in 2.5% phosphoric acid (Sigma), in a 96-well microplate at room temperature. After 10 min, the absorbance was measured at 540 nm using a microplate reader (Tecan), and nitrite concentrations were calculated based on the standard curve generated with sodium nitrite (Sigma), ranging from 0 to 20 μg/ml.

### Blocking test using antibodies against TLRs

To assess the functional role of TLR2 or TLR4 in cytokine production, RAW264.7 cells (5.0 × 10^5^ cells/ml) were incubated with either 50 μg/ml polyclonal anti-TLR2 or 20 μg/ml polyclonal anti-TLR4 (eBioscience, San Diego, CA) in a 24-well plate for 1 h at 37°C, and then 1 μg/ml BoNT/A was added to the cells. Mouse anti-IgG1 (20 μg/ml) and rat anti-IgG2a (20 μg/ml) were used as negative controls, respectively. After incubation for 24 h, culture supernatants were collected and assayed for TNFα and NO.

### Blocking test using NF-κB and MAPK inhibitors

To assess the functional role of the JNK, ERK, p38, and NF-κB signaling pathways in NO and TNFα production induced by BoNT/A, specific inhibitors of each pathway were used. RAW264.7 cells (5.0 × 10^5^ cells/ml) were incubated with 20 μM SP600125 (JNK inhibitor), 20 μM PD98059 (ERK inhibitor), 20 μM SB203580 (p38 inhibitor), 20 μM SN50 (NF-κB inhibitor) (Calbiochem Biosciences, La Jolla, CA), or their combinations for 1 h at 37°C, and then the pretreated RAW264.7 cells were stimulated with 1 nM BoNT/A. After 24 h, culture supernatants were collected and assayed for TNFα and NO.

### Western blot

Cell lysates from BoNT/A-stimulated RAW264.7 cells were prepared using CelLytic-M mammalian cell lysis/extraction reagent (Sigma) supplemented with a protease inhibitor cocktail (Sigma) 0, 5, 10, 20, 30, 40, 50, and 60 min after 1 nM BoNT/A stimulation or 15 min after 1 μg/ml LPS stimulation with or without 1 nM BoNT/A pretreatment. Lysates were then centrifuged at 10,000 × *g* for 15 min at 4°C and collected for further analysis. Protein concentration was determined using the BCA protein assay kit (Pierce). Protein (50 μg) was run on a 12% SDS-polyacrylamide gel (Invitrogen) and then transferred to a nitrocellulose membrane (Bio-Rad, Hercules, CA). The membranes were blocked with 5% skim milk and incubated overnight at 4°C in primary antibodies (1:1,000) recognizing phospho-p44/42 MAPK, p44/42 MAPK, phospho-p38, p38, phospho-SAPK/JNK, and SAPK/JNK (Cell Signaling, Danvers, MA). The blots were incubated with horseradish peroxidase–conjugated anti-rabbit IgG, (1:2,000) (Cell Signaling) and developed using a western blot detection system (Intron, Seongnam, Korea) according to the manufacturer’s instructions.

### Statistical Analysis

Results are expressed throughout as the means ± standard deviation (SD).

## Results

### Transcriptional profiles of BoNT/A-stimulated RAW264.7 cells

**Fig 1:**
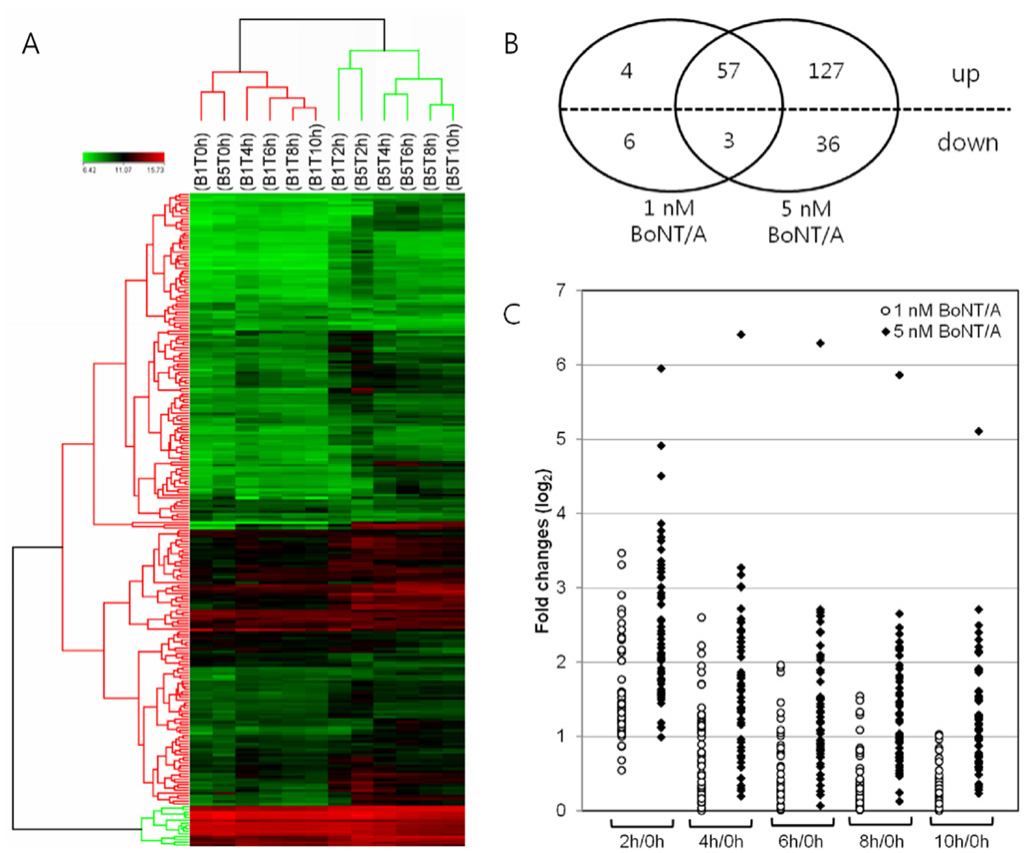
Microarray data analysis. (A) Hierarchical clustering (performed using ArrayAssist) shows subsets of RAW264.7 macrophage genes that are specifically regulated in response to 1 or 5 nM BoNT/A over time (0 to 10 h). Bright green indicates very low signal values, bright red represents very high signal values, and black represents OR median signal values. B1T and B5T represent the concentration of BoNT/A added to RAW264.7 cells (1 or 5 nM), respectively. (B) Venn diagram showing up- and down-regulated genes after 1 or 5 nM BoNT/A. The intersections of the circles indicate the number of genes in common between treatments. The dotted horizontal line separates the up- and down-regulated genes. The cutoffs used were a fold change of at least 2.0 and an adjusted *p* value of <0.05. (C) Time-dependent gene expression changes in BoNT/A-stimulated RAW264.7 cells. Fold changes of 60 genes commonly induced by 1 and 5 nM BoNT/A were recorded according to treatment time.

RAW264.7 cells were treated with two different concentrations (1 and 5 nM) of BoNT/A for 0, 2, 4, 6, 8, and 10 h, and the RNA was isolated and applied to Mouse-6 Expression Beadchips. The time-course experiments were performed in triplicate, and six samples from each independent experiment were hybridized to a single chip to reduce the possibility of chip-to-chip error. Each chip contains six whole-genome gene expression arrays that each represent 46,657 murine transcripts. The array data were processed with the Illumina BeadStudio software program, with data filtering by detecting a *p* value <0.05 and logarithm normalizing with the quantile method (Fig 1A). From the processing procedure, 15,842 probes (33.95%) were selected, and their signal values were analyzed statistically. Gene activation in BoNT/A-treated macrophages was demonstrated by comparison with basal expression levels in RAW264.7 cells at 0 h. A change in gene expression was considered significant if the *p* value of the LPE test was less than 0.05, the fold change was at least 2.0, and an alteration in gene expression occurred in all three independent experiments. For RAW264.7 cells treated with 1 nM BoNT/A, 70 genes (81 probes) displayed statistically significant changes in expression; 61 genes were up-regulated, and nine genes were down-regulated (Fig 1B). In RAW264.7 cells treated with 5 nM BoNT/A, 223 genes (249 probes) showed significant transcriptional changes, including 184 that were up-regulated and 39 that were down-regulated (Fig 1B). Among these genes, 60 genes (70 probes) were induced in response to both concentrations of BoNT/A, whereas 173 genes (1 nM BoNT/A, Table A in S1 File) and 163 genes (5 nM BoNT/A, Table B in S1 File) were differentially expressed depending on the BoNT/A concentration. Computational hierarchical and *k*-means clustering with complete linkage and Euclidean distance (obtained using ArrayAssist) of the normalized signal values corroborated the statistical analyses. Overall, the samples clustered according to BoNT/A concentrations, but each pair of 2-h time point samples clustered together regardless of BoNT/A concentration (Fig 1A). In *k*-means clustering, most of the genes that overlapped with 1 and 5 nM BoNT/A stimulation (58 of 60 genes) showed similar patterns of expression according to the duration of treatment, and more than 96% (55 genes) of the 57 commonly up-regulated genes reached a peak within 4 h after BoNT/A stimulation. Also, as expected, the expression levels of most genes were significantly higher in 5 nM BoNT/A–stimulated macrophages than 1 nM BoNT/A–treated cells (*p*<0.05) (Fig 1C).

### Real-time PCR validation

We employed real-time PCR to confirm the expression of seven up-regulated genes including *tlr2*, *tnf*, *inos*, *ccl4*, *slpi*, *stx11*, and *irg1*, which were chosen among the differentially expressed genes (DEGs) based on their known involvement in inflammation, signaling, or vesicle transport in neurons. Real-time PCR for each target gene was performed using samples treated for 2, 4, 6, 8, and 10 h with 1 and 5 nM BoNT/A. As shown in Table 2, quantitative PCR analysis of expression of these seven genes showed a positive correlation with our microarray expression data (*r* ^*2*^ = 0.8389–0.9786), although the fold increase of these genes detected with real-time PCR tended to be higher than that detected in the DNA microarray analysis.

**Table 2:** Validation by real-time RT-PCR of selected genes determined to be upregulated by treatment of RAW264.7 macrophage cells with 1 nM or 5 nM BoNT/A for 2, 4, 6, 8, and 10 h.

GenBank accession no.	Gene	BoNT/A	Fold change^*a*^ as determined by:										Correlation between microarray and real-time PCR data (*r* ^*2*^)
			Microarray					qRT-PCR					
			2h	4h	6h	8h	10h	2h	4h	6h	8h	10h	
NM_011905.2	*tlr2*	1 nM	2.13	2.21	1.50	1.29	1.13	3.49	2.88	2.39	1.73	1.28	0.8389
		5 nM	3.57	3.35	1.96	1.54	1.18	5.55	3.72	1.88	1.27	0.92	
NM_013693	*tnf*	1 nM	4.53	1.88	1.69	1.79	1.69	6.31	2.09	2.17	1.94	2.02	0.9786
		5 nM	8.24	5.77	6.56	5.50	4.90	12.23	8.14	8.78	6.63	5.71	
NM_010927.1	*inos*	1 nM	1.31	1.79	1.50	1.45	1.33	2.68	5.67	4.46	3.78	3.89	0.9559
		5 nM	3.74	4.85	4.85	3.95	3.66	9.29	11.72	11.14	8.87	7.48	
NM_013652	*ccl4*	1 nM	4.05	1.37	1.11	1.16	1.37	5.69	1.37	1.11	1.12	1.35	0.9179
		5 nM	10.21	6.00	5.36	4.66	5.37	31.03	11.82	10.09	8.49	8.20	
NM_011414.1	*slpi*	1 nM	1.04	1.09	1.07	1.08	1.12	0.87	0.72	1.05	1.14	1.22	0.9272
		5 nM	1.51	2.00	2.82	3.27	3.67	0.74	1.54	5.32	8.03	8.89	
XM_203312.2	*Stx11*	1 nM	2.56	1.52	1.10	1.07	1.04	10.29	4.16	2.11	1.41	1.30	0.9582
		5 nM	3.74	1.81	1.34	1.39	1.58	25.78	8.15	3.45	3.37	3.80	
XM_127883	*irg1*	1 nM	6.36	7.57	5.05	3.36	2.53	27.78	28.04	20.96	11.14	5.66	0.9531
		5 nM	44.90	58.17	54.20	42.73	29.11	319.27	38.77	312.20	198.97	118.41	

^*a*^The values shown are means of three independent experiments.

### Identification of biological processes and signaling pathways modulated by BoNT/A

**Fig 2:**
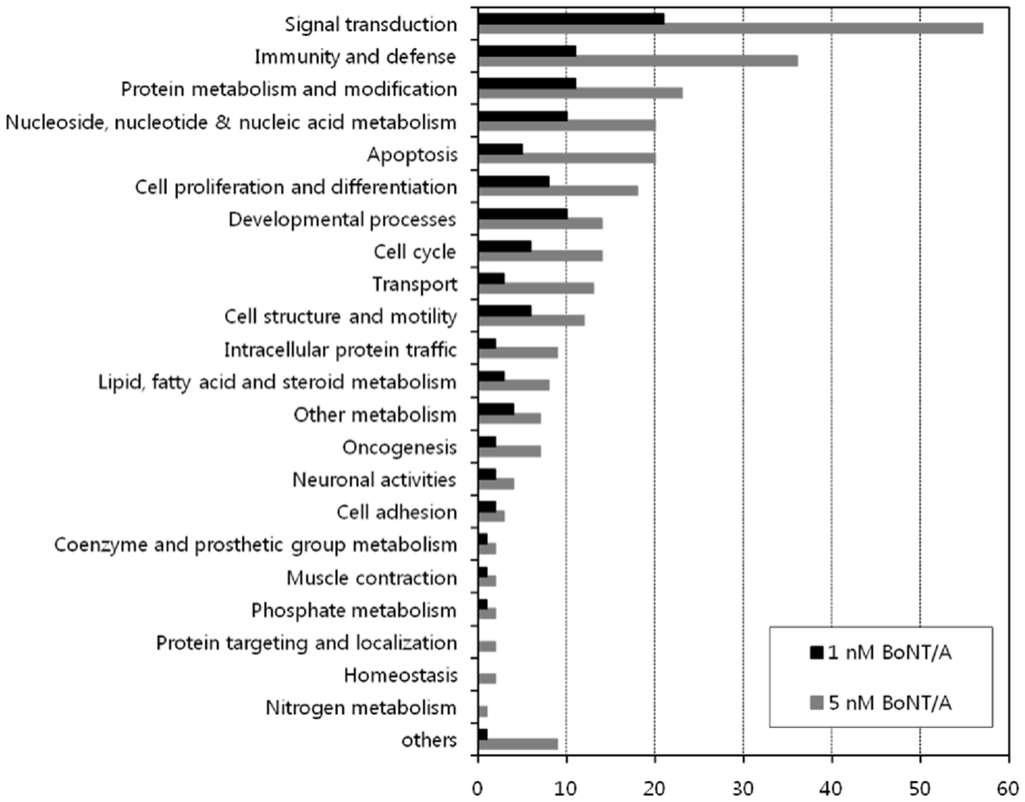
Functional classification of DEGs after the stimulation of RAW264.7 macrophage-like cells with BoNT/A (1 or 5 nM). Each bar indicates the absolute number of down- or up-regulated genes identified within each functional class.

DEGs between 0 h and other times following BoNT/A treatment of the macrophages were analyzed using the Panther database to identify altered biological processes (Fig 2). In cells stimulated with both 1 and 5 nM BoNT/A, Panther classification of the 233 DEGs showed that a broad range of major functional processes was affected. A marked effect was found in biological processes involving signal transduction and immunity/defense, in which 30.9% of DEGs (72 of 233 genes) were affected. Considering the clinical characteristics of BoNT/A, we were interested in determining if the DEGs were associated with neuronal activities, intracellular protein trafficking, and muscle contraction. Compared to cells treated with 1 nM BoNT/A, cells treated with 5 nM BoNT/A showed changes in nine biological processes that were over-represented including homeostasis, protein targeting and localization, nitrogen metabolism, blood circulation and gas exchange, sensory perception, electron transport, carbohydrate metabolism, sulfur metabolism, and non-vertebrate processes. Table A and B in S1 File list the DEGs based on grouping according to their known or proposed biological function.

Signaling pathways of cellular processes induced by BoNT/A were also analyzed using both the David database (KEGG) and the PathwayArchitect database (Table 3). Although there was a difference in the number of related probes between KEGG and PathwayArchitect due to analysis theory, both analyses showed involvement of DEGs in cytokine-cytokine receptor interactions, TLR, MAPK signaling pathways, apoptosis, and the p53 signaling pathway. In addition, signaling pathways relevant to the cell cycle, prostate cancer, glioma, and the hematopoietic cell lineage were observed with KEGG analysis, whereas pathways related to SAPK-JNK signaling, asthma, p38 signaling, and mitochondrial apoptosis control were seen with PathwayArchitect analysis.

**Table 3:** Representative signaling pathways of BoNT/A-derived cellular mechanisms using David database (KEGG) and PathwayArchitect database.

Involvement between KEGG / PathwayArchitect	Database			
	KEGG		PathwayArchitect	
	Pathways	Probe number	Pathways	Probe number
Common	Cytokine-cytokine receptor interaction	15	TNF signaling	36
			IL-1 and IL-6 signaling	40
	MAPK signaling pathway	11	MAPK signaling	102
	Toll-like receptor signaling pathway	9	Toll-like receptor	82
	Apoptosis	4	Apoptosis	144
	P53 signaling pathway	4	P53 signaling	67
Others	Cell cycle	6	SAPK-JNK signaling	130
	Prostate cancer	5	Asthma	122
	Glioma	4	P38 signaling	105
	Adipocytokine signaling pathway	4	Mitochondrial apoptosis control	100
	Hematopoietic cell lineage	4	NF-κB signaling	81
	Chronic myeloid leukemia	4	FAS signaling	80

### BoNT/A induces genes relevant to signal transduction and immunity/defense

In BoNT/A-treated RAW264.7 cells, the most remarkable finding was the number of DEGs involved in signal transduction. Thirty percent (21 of 70) and 25.6% (57 of 223) DEGs belonged to this category in 1 and 5 nM BoNT/A-treated RAW264.7 cells, respectively. In 1 nM BoNT/A-treated cells, 20 genes were up-regulated, and only 1 gene was down-regulated, whereas in 5 nM BoNT/A-treated cells, 50 genes were up-regulated, and 7 genes were down-regulated. These genes included those encoding cell surface receptors or receptor–mediated proteins (e.g., TNFRSF, RGS1, TLR2, IRAK2, ADORA2b, EDN1, CCRL2, TRAF1), intracellular signaling cascade-associated proteins (e.g., MLP, PLK, SOCS3, iNOS), and signaling molecules participating in cell communication (e.g., CCL4, TNF, IL1b, CSF3, GP49a, CXCL2). Several of these genes are ultimately linked with immune responses through their signal transduction pathways. Among the 37 DEGs in the immune/defense category, 22 DEGs (59.5%) overlapped with the signal transduction category.

### Effects of BoNT/A on macrophage NO/cytokine expression

**Fig 3:**
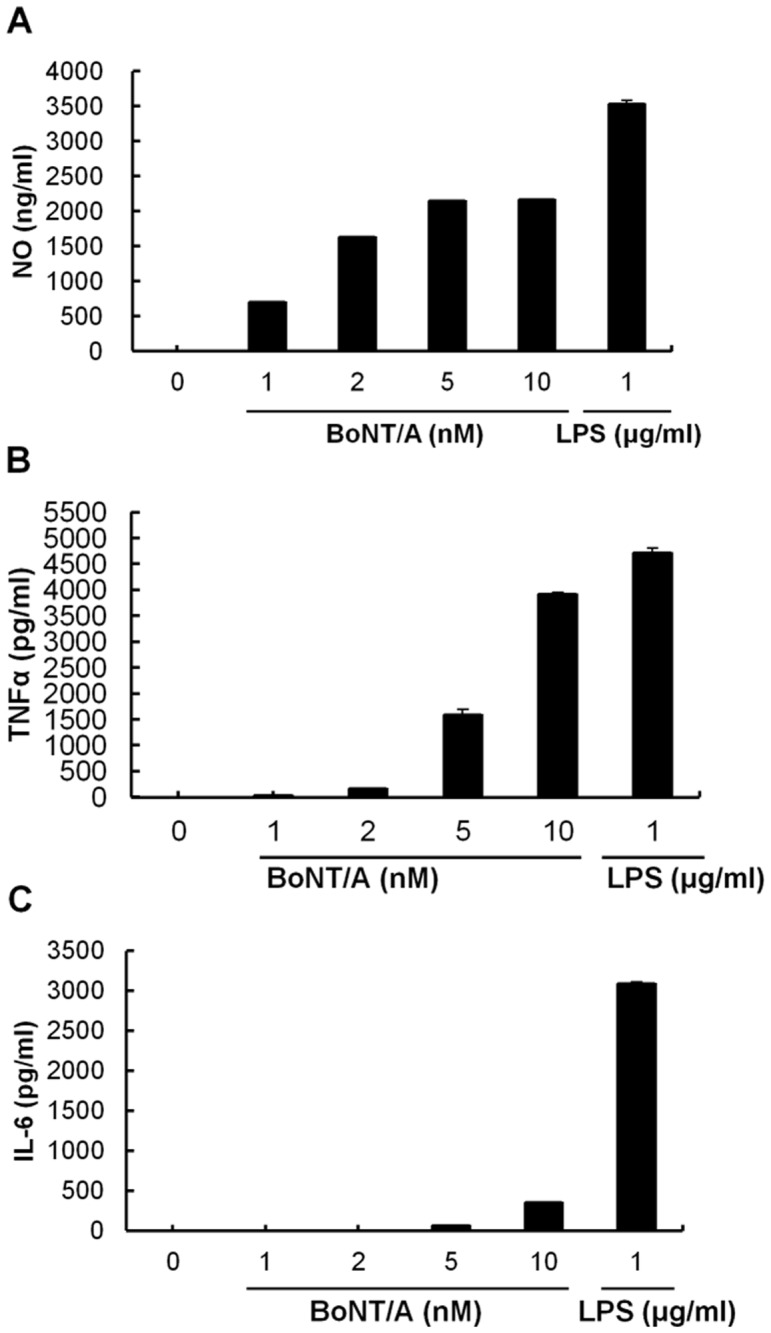
Dose-dependent production of NO (A), TNFα (B), and IL-6 (C) in BoNT/A-treated RAW264.7 cells. Cells were incubated with BoNT/A (0, 1, 2, 5, and 10 nM) or 1 μg/ml LPS as a positive control for 24 h at 37°C. Culture supernatants were collected, and the levels of NO, TNFα, and IL-6 were measured. Values are the mean ± SD of three replicates for each group.

The inflammatory response of RAW264.7 macrophages to various concentrations of BoNT/A (ranging from 0 to 10 nM) was investigated. Following exposure to BoNT/A, RAW264.7 cells expressed increased levels of TNFα and NO in a dose-dependent manner (Fig 3A and 3B). IL-6 was detected only when stimulated with more than 5 nM BoNT/A (Fig 3C). However, IL-1β and IL-12 were not detected with the highest concentration of BoNT/A tested (10 nM). No cytotoxicity was observed with any of the BoNT/A concentrations examined.

### Functional role of TLR2 and the major intracellular MAPK signaling pathways in BoNT/A-induced inflammatory responses

**Fig 4:**
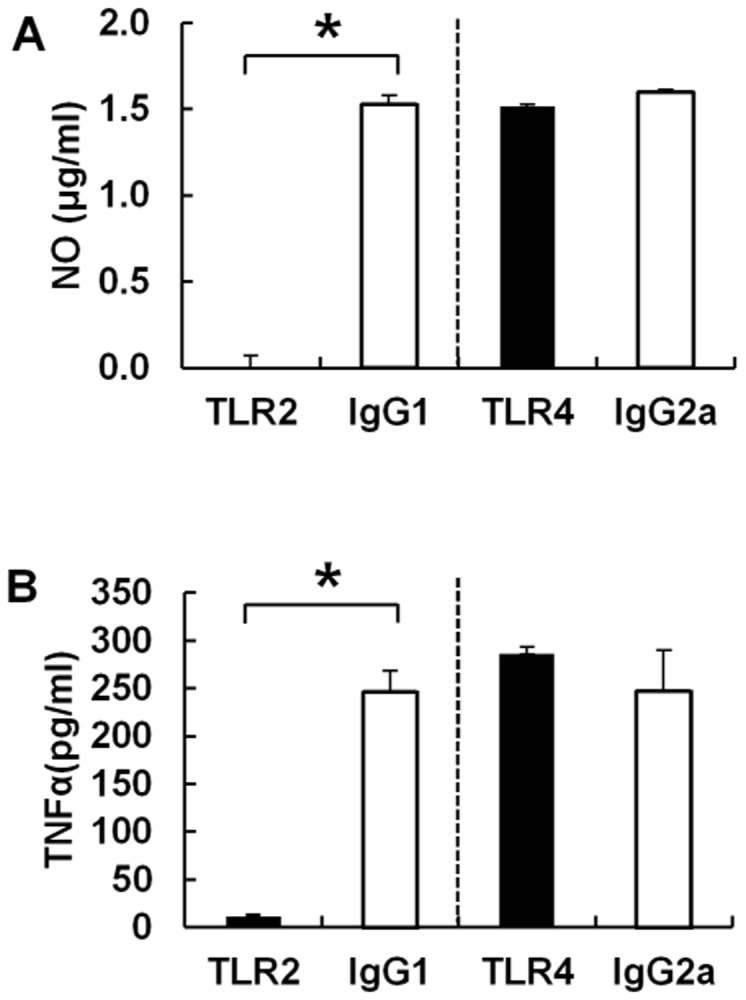
BoNT/A used TLR2 for NO (A) and TNFα (B) production in the RAW264.7 mouse macrophage cell line. The levels of NO (A) and TNFα (B) were assessed in RAW264.7 cells stimulated with BoNT/A after preincubation with polyclonal anti-TLR2 (50 μg/ml) or anti-TLR4 (20 μg/ml). TLR2, TLR4, IgG1, and IgG2a indicate the counterpart antigen against which the respective antibody was used to pretreat RAW264.7 cells. Mouse anti-IgG1 or rat anti-IgG2a was used as a negative control. Anti-TLR2 had a significant (*p*<0.05) inhibitory effect on both NO and TNFα production by RAW264.7 cells stimulated with BoNT/A but anti-TLR4 had no noticeable effect.

TLRs are a set of innate immune receptors that recognize structures common to many different pathogens. TLR-mediated stimulation induces production of pro-inflammatory and immune-related cytokines. Thus, we hypothesized that RAW264.7 cells would utilize TLR in response to BoNT/A, resulting in induction of NO and TNFα. To investigate the functional involvement of TLR in BoNT/A-induced NO and TNFα responses by RAW264.7 cells, the cells were incubated with polyclonal anti-TLR2 (50 μg/ml) or anti-TLR4 (20 μg/ml) before stimulation with BoNT/A. Compared to the negative control that was incubated with mouse anti-IgG1 or rat anti-IgG2a, anti-TLR2 almost completely blocked both NO (Fig 4A left) and TNFα (Fig 4B left) production from BoNT/A-stimulated RAW264.7 cells (*p*<0.05), whereas anti-TLR4 had no noticeable effect on NO (Fig 4A right) and TNFα (Fig 4B right) production. These results demonstrate that TLR2, but not TLR4, is essential for the induction of NO and TNFα by murine macrophages stimulated with BoNT/A.

**Fig 5:**
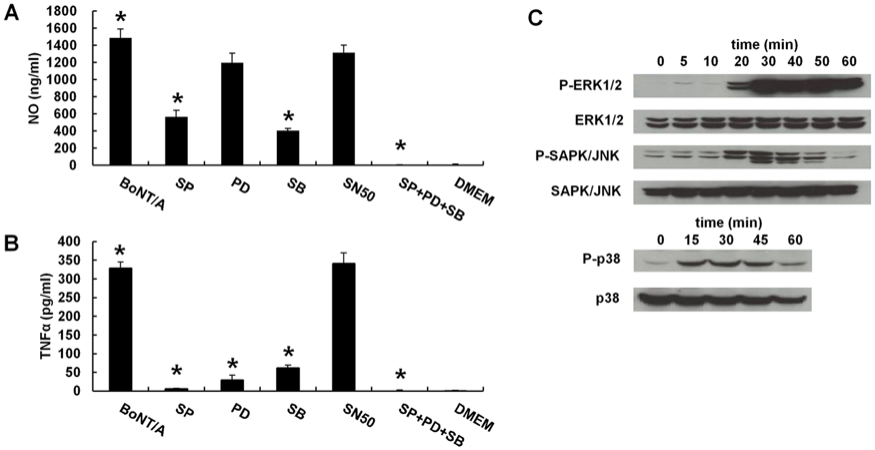
BoNT/A induced phosphorylation of MAPK molecules in RAW264.7 cells. A and B, production of NO (A) and TNFα (B) following stimulation with BoNT/A (1 nM) in RAW264.7 cells pretreated with specific inhibitors of each pathway. RAW264.7 cells (5.0 × 10^5^ cells/ml) were incubated with 20 μM SAPK/JNK inhibitor (SP), 20 μM ERK inhibitor PD98059 (PD), 20 μM p38 inhibitor (SB), 20 μM NF-κB inhibitor (SN50), or their combinations for 1 h at 37°C and then stimulated with 1 nM BoNT/A for 24 h. The levels of NO (A) and TNFα (B) production were assessed in the supernatants. C, western blot of phosphorylation of the signaling molecules in BoNT/A-stimulated RAW264.7 cells. The cells were incubated with 1 nM BoNT/A for 0 to 60 min. The proteins from the cells were used to detect phosphorylated or total forms of NF-κB or the three MAPK molecules. Representative results of three independent experiments are shown.

We also examined the TLR-mediated intracellular signaling pathways in BoNT/A-stimulated RAW264.7 cells. The pathways were analyzed with a blocking test using inhibitors of NF-κB and three MAPK molecules including ERK1/2, JNK, and p38 MAPK. RAW264.7 cells were treated with the inhibitor prior to stimulation with 1 nM BoNT/A for 24 h, and then the concentration of NO and TNFα was determined in the supernatants. The p38 inhibitor and the JNK inhibitor (SP600125) each significantly reduced BoNT/A-induced NO and TNFα production in RAW264.7 cells (*p*<0.05). The ERK inhibitor effectively suppressed TNFα production in BoNT/A-stimulated RAW264.7 cells (*p*<0.05) but only slightly decreased NO production. The combination of p38, JNK, and ERK inhibitors completely blocked NO and TNFα production. However, the NF-κB inhibitor did not block NO or TNFα production in BoNT/A-stimulated RAW264.7 cells (Fig 5). These results were confirmed by examining phosphorylation of the signaling molecules. Activation of MAPKs is dependent on phosphorylation by their respective upstream MAP kinases. Thus, MAPK phosphorylation was analyzed with western blot analysis using phospho-specific antibodies. RAW264.7 cells were treated with 1 nM BoNT/A for 0 to 60 min. As shown in Fig 5, phosphorylation of all three MAPKs occurred within 20 min of BoNT/A stimulation and peaked at 30 min. These results indicate that BoNT/A-stimulated RAW264.7 cells induce NO and TNFα production through TLR2-mediated signal transduction via activation of ERK, JNK, and p38.

### BoNT/A inhibits the production of NO and proinflammatory cytokines in LPS-stimulated RAW264.7 cells

**Fig 6:**
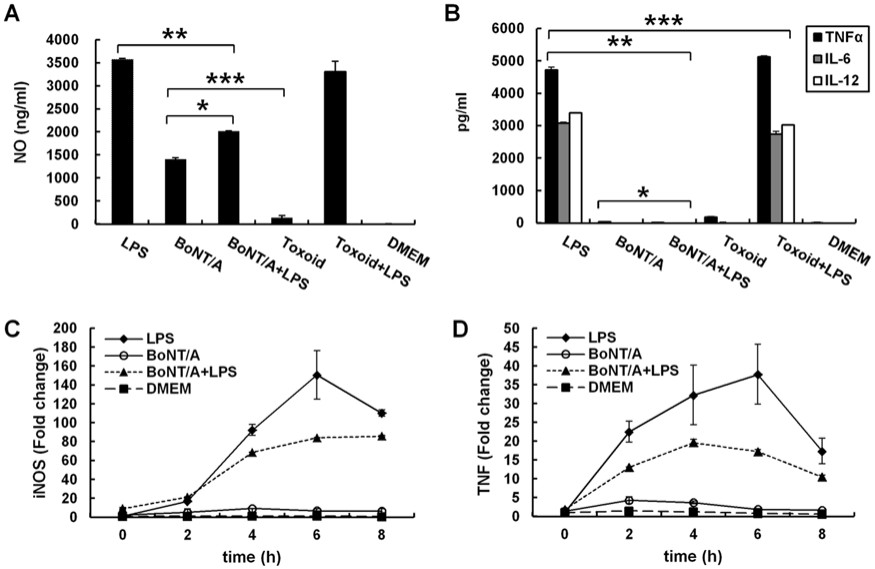
BoNT/A inhibited the production of LPS-induced pro-inflammatory mediators in RAW264.7 cells. A and B, production of NO and proinflammatory cytokines in LPS-stimulated RAW264.7 cells with or without BoNT/A pretreatment. RAW264.7 cells were pretreated with 1 nM BoNT/A or 1 nM BoNToxoid/A for 24 h, and then stimulated with or without 1 μg/ml LPS. Culture media were collected at 24 h to measure NO (A), TNFα, IL-6, and IL-12 (B) concentrations using the Griess reaction and sandwich ELISA, respectively. Three independent experiments were performed, and the data are the mean ± S.D. *, *p*<0.05 vs. LPS alone. C and D, quantification of mRNA expression of *Tnf* and iNOS in LPS-stimulated RAW264.7 macrophages with or without BoNT/A pretreatment. Total RNAs were isolated, and mRNA levels of iNOS and TNFα were analyzed with qRT-PCR. GAPDH expression was used as an internal control for RT-PCR. Representative results of three independent experiments are shown.

To further investigate whether BoNT/A modulates the production of NO and proinflammatory cytokines, we treated RAW264.7 cells with 1 nM BoNT/A with or without 1 μg/ml LPS and analyzed culture supernatants for NO and cytokines. RAW264.7 cells expressed significant levels of NO, TNFα, IL-6, and IL-12 upon exposure to LPS (Fig 6A and 6B). Parallel cultures of RAW264.7 cells were exposed to BoNT/A or BoNToxoid/A. Upon exposure to BoNT/A, RAW264.7 cells induced low levels of NO compared to LPS-exposed cells. TNFα was expressed at very low levels, whereas IL-6 and IL-12 were not detected. BoNToxoid/A-treated cells produced barely detectable levels of NO and three proinflammatory cytokines. Importantly, pretreatment of RAW264.7 cells with BoNT/A and subsequent addition of LPS markedly decreased NO production and almost completely blocked the expression of TNFα, IL-6, and IL-12 from RAW264.7 cells (Fig 6A and 6B).

**Fig 7:**
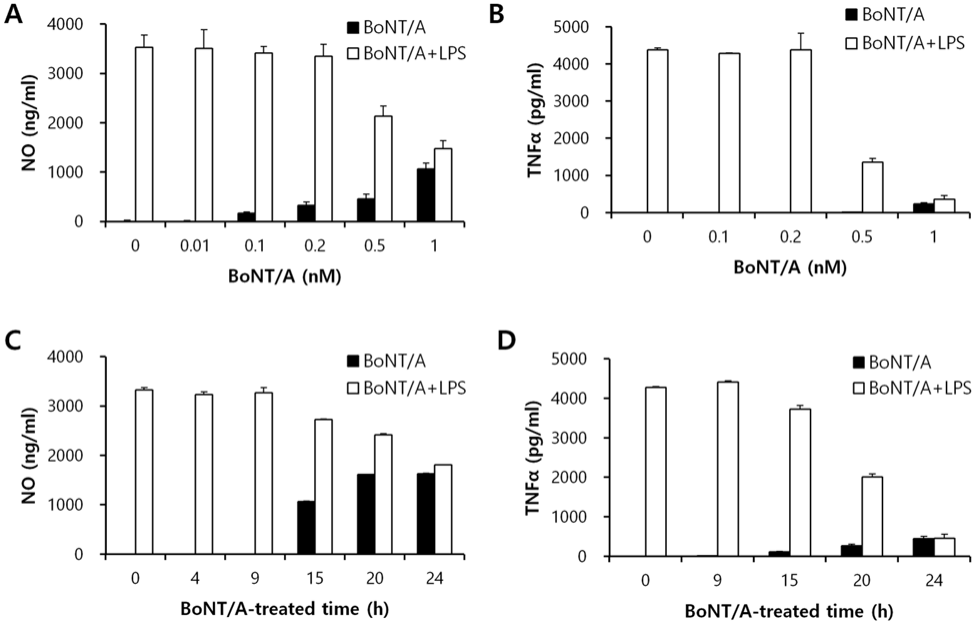
BoNT/A inhibited LPS-induced production of TNFα and NO in a dose- and time-dependent manner in RAW264.7 macrophages. RAW264.7 cells were treated with 0 to 1 nM BoNT/A for 24 h (A and B) or with 1 nM BoNT/A for 0 to 24 h and then stimulated with or without 1 μg/ml LPS (C and D). Culture media were collected at 24 h to measure NO (A and C) and TNFα (B and D) concentrations using the Griess reaction and sandwich ELISA, respectively. Three independent experiments were performed, and the data shown are the mean ± SD.

Preincubating macrophages with various concentrations of BoNT/A (0 to 5 nM) over time (0 to 32 h) progressively inhibited the ability of the cells to produce NO and TNFα upon subsequent exposure to LPS. The inhibitory effect by BoNT/A occurred in a dose-dependent manner, and 2 nM and 1 nM BoNT/A was required for complete inhibition of NO and TNFα production, respectively (Fig 7A and 7B). With exposure to 1 nM BoNT/A, inhibition of NO and TNFα became apparent after 15 h of exposure and reached a maximum by 24 h (Fig 7C and 7D). However, co-incubating the cells with BoNT/A and LPS was insufficient to inhibit cytokine expression (Fig 6A and 6B).

To further understand the inhibitory effects of BoNT/A on LPS-induced expression, iNOS and TNF mRNA levels were investigated using qRT-PCR. Culturing the cells with BoNT/A alone induced transcription of iNOS and TNFα at detectable but low levels compared to LPS, whereas LPS induced strong iNOS and TNFα mRNA expression. Preincubation with BoNT/A significantly inhibited the LPS-induced transcription, and the inhibitory effect appeared strongest at 4 h (Fig 6C and 6D). These data indicate that BoNT/A suppresses the expression of iNOS and TNFα at the transcriptional level in LPS-stimulated RAW264.7 cells.

### BoNT/A suppresses the phosphorylation of MAPKs in LPS-stimulated RAW264.7 cells

**Fig 8:**
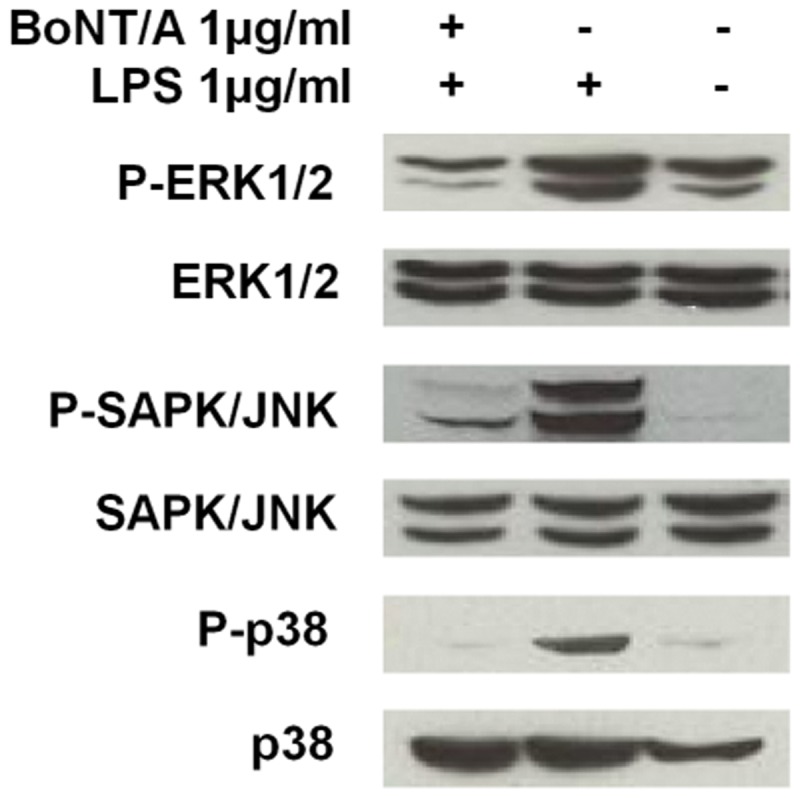
BoNT/A suppressed the phosphorylation of MAPKs in LPS-stimulated RAW264.7 macrophages. RAW264.7 cells were pretreated with 1 nM BoNT/A for 24 h, and then stimulated with 1 μg/ml LPS for 24 h. The cellular proteins were used to detect phosphorylated or total forms of the three MAPKs, ERK1/2, JNK1/2, and p38. Representative results of three independent experiments are shown.

MAPKs (including ERK, JNK/SAPK, and p38) are important regulators of iNOS-NO expression by IFNγ and LPS [27]. Thus, MAPKs are likely to be associated with the anti-inflammatory effects of BoNT/A in LPS-stimulated RAW264.7 cells. RAW264.7 cells were stimulated with 1 μg/ml LPS for 15 min with or without 1 nM BoNT/A, and the cell lysates were then used to examine the phosphorylation of MAPKs with western blotting. LPS stimulation strongly induced the phosphorylation of ERK, JNK, and p38 in RAW264.7 cells (Fig 8). However, BoNT/A significantly suppressed the phosphorylation of these three MAPK molecules, whereas the non-phosphorylated forms of these MAPKs remained unchanged. Untreated RAW264.7 cells expressed basal levels of ERK, JNK, and p38. These results indicate that signal transduction by MAPKs may be effectively blocked by BoNT/A in activated macrophages.

### TLR2 -/- KO mice show TLR2 specific inflammatory responses in BMDM cells

**Fig 9:**
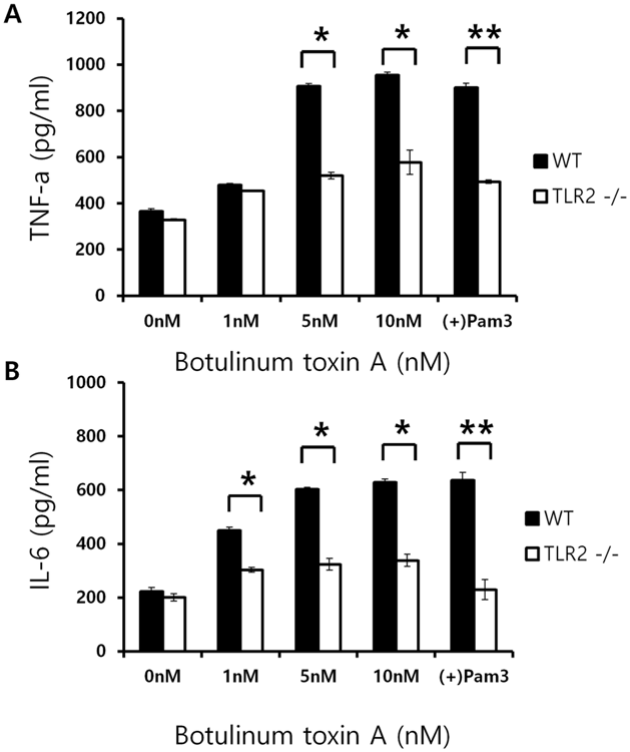
TLR2 KO mice show decreased expression of TNFα and IL-6 cytokines upon BoNT/A stimulation. WT (black bar; n = 6) and TLR2 KO mice (white bar; n = 6) were used to isolate bone marrow monocytes. Differentiated BMDM cells and subjected to BoNT/A treatment. Culture supernatants were analyzed for TNFα (A) and IL6 (B). All data were given as means ± SD.

To confirm the specific involvement of TLR2 in BoNT/A-induced TNFα and IL-6 responses by primary macrophage cells, the BMDM cells were generated from both of wild type and TLR2 KO mice. Compared to the negative control (0 nM) and positive control (Pam3), BoNT/A mediated TNFα (Fig 9A) and IL-6 (Fig 9B) responses were almost completely abolished in BMDM primary cells from TLR2-/- Knock out mice. These results confirm that TLR2 is specific for the induction of TNFα and IL-6 by macrophages *in vivo* stimulated with BoNT/A.

## Discussion

BoNT/A classically causes botulism, which is characterized by fatal flaccid muscle paralysis. The majority of natural botulism cases occurs when adults and animals ingest preformed botulinum toxin in contaminated foods [1] or when infants uptake *C*. *botulinum* pathogens. The ingested toxins cause paralysis by blocking neurotransmitter release at the neuromuscular junction. To date, application of BoNT/A has been extended to the fields of therapeutics and biodefense [28]. Approaches to using BoNT/A as a therapeutic drug have been extensively reported, and the toxin has led to new treatments for neurological and endocrine disorders such as cerebral palsy, Parkinson’s disease, Graves’ disease, and Cushing’s disease [29]. Simultaneously, its possible use as a biological weapon has also been discussed due to the lethal characteristics, with less than one microgram sufficient to cause fatal human disease. In a bioterrorism attack, the routes of exposure would most likely be oral or inhalation [4]. However, there is little reference in the literature to the cellular effects or other actions that the toxins may have on host immune cells. However, immunological research on BoNT is essential for developing advanced vaccines, research on biodefense, and countermeasures to the side effects from therapeutic applications.

In this study, we show the global transcriptional responses of host immune cells to BoNT/A and how these changes in gene regulation affect macrophage function. The genes affected by BoNT/A treatment fell into the following functional categories (listed in order of decreasing frequency): signal transduction, immunity and defense, protein metabolism and modification, nucleic acid metabolism, apoptosis, cell proliferation and differentiation, and others. Among these categories, signal transduction and immunity/defense comprised 30.9% (72 of 233 genes) of the DEGs. Signaling due to BoNT/A-derived cellular mechanisms was predominantly associated with cytokine-cytokine receptor interactions, TLRs, MAP signaling pathways, apoptosis, and the p53 signaling pathway. Thus, RAW264.7 macrophages recognize and respond to BoNT/A through various signaling pathways that are involved in immune reactions. Upon infection, macrophages recognize pathogen-specific molecular patterns through TLRs, and TLR signaling stimulates macrophage activation by inducing production of proinflammatory cytokines and NO, followed by microbial uptake via phagocytosis [16]. TLR2 also induces expression of other immunoregulatory genes such as RGS1 and SOCS3.

Among all RGS proteins tested, only RGS1 and RGS2 are modulated by bacterial lipopeptides (TLR2/1 or TLR2/6 ligands) and LPS (TLR4/MD2 ligand). RGS1 mRNA was up-regulated during the first 30 min after stimulation, followed by down-regulation [30]. Similarly, we detected rapid and transient up-regulation of *Rgs1* mRNA in RAW264.7 cells treated with both 1 and 5 nM BoNT/A, indicating a possible relationship between BoNT/A and TLR-mediated RGS1 expression.

Like RGS1, suppressor of cytokine signaling 3 (SOCS3) is also up-regulated by LPS or TLR stimulation in macrophages [31,32]. SOCS3 then mediates feedback inhibition of the macrophage activation by negatively regulating cytokine signaling. SOCS3 accomplishes this by preventing JAK-mediated activation of STAT3, inhibiting the NF-κB pathway, antagonizing cAMP-mediated signaling, and enhancing signaling through the MAPK pathway [33,34]. On the other hand, induction of SOCS3 can also be used by microbes to evade immune defense, an action that is exemplified by *Mycobacterium bovis BCG* and the parasite *Toxoplasma gondii* [32,35]. We observed that 2 h after BoNT/A stimulation, the *Socs3* expression level (12.86-fold) was higher in comparison with other genes, and it gradually decreased over 10 h (4.44-fold). Because SOCS3 expression is increased at sites of ongoing inflammation [34], our data indicate that not only BoNT/A-stimulated RAW264.7 cells, but BMDM cells evoke inflammatory responses.

From another perspective, our data also suggest that BoNT/A may act as an immune stimulus, although the response from immune cells was not strong compared with LPS. In fact, BoNT/A induced the production of NO, TNFα, and IL-6 from RAW264.7 cells, as shown by our data demonstrating up-regulation of proinflammatory mediators at translational and transcriptional levels. Other types of BoNT also induce proinflammatory mediators. BoNT/B increases the production of IL-6 from splenic lymphocytes, and BoNT/D induces production of TNFα and NO from human monocytes [36,37].

LPS is the best characterized immune stimulatory molecule in gram-negative bacteria. LPS can provoke a variety of immunostimulatory responses, for example, production of proinflammatory cytokines such as TNFα, IL-1β, IFN-γ, IL-6, and IL-12 and inflammatory effector substances such as NO. Our results also clearly showed up-regulation of proinflammatory genes encoding iNOS and TNFα and the production of NO, TNFα, IL-6, and IL-12 in response to LPS stimulation in RAW264.7 macrophages. The effects of LPS have been extensively described in the literature [38]. Although the innate immune response is critical for controlling the growth of pathogenic microorganisms, excessive inflammatory cytokine production is harmful to the host and can even result in septic shock, which can be fatal. Therefore, inhibitors of these inflammatory molecules have been considered as candidate anti-inflammatory drugs.

In this study, we showed that BoNT/A efficiently inhibited the production of TNFα and NO by suppressing their mRNA expression in LPS-stimulated RAW264.7 cells in a dose- and time-dependent manner. However, simultaneous treatment with BoNT/A and LPS did not inhibit macrophage production of NO and TNFα in response to LPS. Thus, pretreatment of RAW264.7 cells with BoNT/A was minimally required to inhibit LPS-induced macrophage responses (see Fig 7). The anti-inflammatory effect of BoNT/A was found for all examined cytokines including IL-6 and IL-12 as well as TNFα and NO. However, BoNToxoid/A did not attenuate the production of proinflammatory mediators (Fig 6A and 6B). From these results, we infer that interaction between active BoNT/A and macrophages is essential for the anti-inflammatory properties of BoNT/A and that the properties associated with negative regulation of LPS-activated signal transduction result in the production of proinflammatory mediators in macrophages.

TLR2 and TLR4 are the best-studied innate immune receptors, and they lead to induction of direct antimicrobial pathways, expression of co-stimulatory molecules, and release of cytokines and chemokines via NF-κB and/or MAPK signaling. In this report, the result of blocking tests using antibodies against TLR revealed that BoNT/A is sensed by TLR2 but not by TLR4, triggering NO and TNFα production in the mouse macrophage cell line. In other words, innate receptors are not related to the anti-inflammatory responses of BoNT/A following LPS exposure because BoNT/A and LPS are recognized by different TLRs [16]. This idea is supported by our array data showing that BoNT/A increased the level of TLR2 gene expression in macrophages in a dose-dependent manner. This is the first report to demonstrate that macrophages utilize TLR2 to detect BoNT/A.

We also identified the signaling mechanism involved in the macrophage response to BoNT/A. BoNT/A induced phosphorylation of ERK, JNK, and p38 in macrophages. Simultaneous treatment with ERK, JNK, and p38 inhibitors completely blocked NO and TNFα production. However, the role of each MAPK was different. p38 and JNK were the most important kinases for NO and TNFα production, respectively. However, ERK played a role in TNFα production but had little effect on NO production. NF-κB was not activated by BoNT/A. Results from analysis of BoNT/A-derived cellular mechanisms from the array data provided additional evidence showing involvement of MAPK signaling in macrophage responses to BoNT/A. Analysis using the David database (KEGG) and the PathwayArchitect database suggested involvement of the MAPK and TLR signaling pathways. The MAPK signaling pathway was also associated with anti-inflammatory responses mediated by BoNT/A following LPS treatment. Pretreatment with BoNT/A attenuated the phosphorylation of ERK, JNK, and p38 in LPS-activated macrophages. These results are consistent with a previous report showing that IFN-γ plus LPS induction of iNOS is modulated by ERK, JNK/SAPK, and p38 in a mouse macrophage cell line [27].

So far, multiple mechanisms have been presented for preventing the harmful effects of the endotoxin [39,40]. First, the expression levels of TLR/MD2 complexes on the macrophage surface are down-regulated after LPS stimulation [41]. Second, the inhibitory molecules of LPS signaling such as SOCS-1, SOCS-3, and IRAK-M, are induced after LPS stimulation and prevent excessive production of proinflammatory cytokines [42–44]. These mechanisms are called LPS tolerance, because they inhibit the response to a second challenge with the endotoxin. Third, LPS-induced shock is prevented by inhibiting the TLR signaling pathway [40]. Microarray and cellular response data provided information to clarify the mechanisms of BoNT/A-mediated modulation and production of proinflammatory mediators. BoNT/A up-regulates expression of genes, such as *socs3*, *lilrb4*, *stx11*, and *slpi*, to modulate inflammatory responses by inhibiting LPS signaling, resulting in reduction of LPS-induced cytokine production [45–50]. Moreover, BoNT/A exerts anti-inflammatory activity by inhibiting MAPK signaling such as through ERK, JNK, and p38. Thus, the anti-inflammatory function of BoNT/A in LPS-stimulated macrophages results from multiple mechanisms modulating expression of proinflammatory mediators. TLR2 plays an important role in host immune response to infections by many different microbial pathogens. BoNT/A can induce a low level of TNF and NO production, and these molecules are almost completely blocked by anti-TLR2, suggesting that TLR2 is a major receptor that macrophages use to recognize BoNT/A. KO mice experiments (Fig 9) also confirm that TLR2-dependent activation by BoNT/A may contribute to efficient innate immunity to BoNT/A intoxication *in vivo*.

In conclusion, our findings provide more insight into the early events in the host response upon exposure to BoNT/A and further understanding of the molecular basis of innate immune cell activation after BoNT/A stimulation. In addition to recent reports [34,51,52], we suggest that BoNT/A induces global gene expression and a host immune response to BoNT/A that proceeds through a TLR2-dependent pathway, which is modulated by JNK, ERK, and p38. These results provide additional critical considerations for therapeutic applications in both biodefense and BoNT medicine.

## Supporting Information

S1 File Figure A. Purity and identification of Botulinum toxin A preparation. Endotoxin free-BoNT/A have been purified by Superdex 200 (A) and affinity chromatography (B) as described in materials and methods. Superdex 200 FPLC preparation showed BoNT/A heavy chain (Hc), light chain (Lc), NTNH, and haemagglutinins on SDS-PAGE analysis. Affinity chromatography showed only Hc and Lc chains with no other contaminants. Hc (C) and Lc (D) were identified by peptide mass fingerprint analysis.
(PDF)
