# Supplementary material for: Botulinum Neurotoxin Type A Induces TLR2-Mediated Inflammatory Responses in Macrophages
Source: PLoS One. 2015 Apr 8;10(4):e0120840. doi: 10.1371/journal.pone.0120840 (PMC4390353; doi:10.1371/journal.pone.0120840)
Supplement: S1 File — Endotoxin free-BoNT/A have been purified by Superdex 200 (A) and affinity chromatography (B) as described in materials and methods. Superdex 200 FPLC preparation showed BoNT/A heavy chain (Hc), light chain (Lc), NTNH, and haemagglutinins on SDS-PAGE analysis. Affinity chromatography showed only Hc and Lc chains with no other contaminants. Hc (C) and Lc (D) were identified by peptide mass fingerprint analysis. (PDF) [file pone.0120840.s001.pdf]

Supplemental Materials

Figure A. Purity and identification of Botulinum toxin A preparation

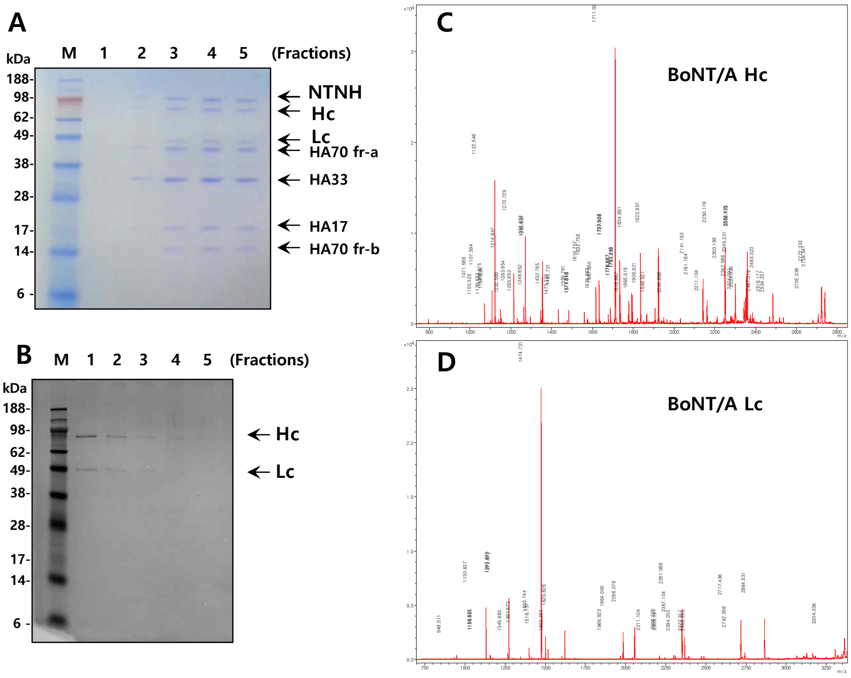

Endotoxin free-BoNT/A have been purified by Superdex 200 (A) and affinity chromatography (B) as described in materials and methods. Superdex 200 FPLC preparation showed BoNT/A heavy chain (Hc), light chain (Lc), NTNH, and haemagglutinins on SDS-PAGE analysis. Affinity chromatography showed only Hc and Lc chains with no other contaminants. Hc (C) and Lc (D) were identified by peptide mass fingerprint analysis.

Table A. Mouse macrophage genes that are differentially expressed in response to 1 nM of BoNT/A according to 2, 4, 6, 8, and 10 h of treatment times compared to 0 h

GenBank ID	Gene symbol	Gene or protein	Fold change (according to treatment time)				
			2 h	4 h	6 h	8 h	10 h
**Signal transduction (36)**							
NM_007707.2	*Socs3*	Suppressor of cytokine signaling 3	2.89	2.08	1.20	-1.03	1.04
NM_009421.2	*Traf1*	Tnf receptor-associated factor 1	4.52	2.24	1.22	1.04	1.19
NM_010090.2	*Dusp2*	dual specificity phosphatase 2	2.78	1.27	1.06	1.01	1.11
NM_176933	*Dusp4*	dual specificity phosphatase 4	3.06	1.22	1.02	1.05	1.22
NM_130447.1	*Dusp16*	dual specificity phosphatase 16	2.97	1.05	-1.20	-1.05	-1.12
NM_013908.1	*Fbxw5*	F-box and WD-40 domain protein 5	-8.80	-9.83	-5.25	-9.74	-1.49
NM_010807.2	*Mlp*	MARCKS-like protein	4.45	4.34	3.65	2.52	1.61
NM_030720.1	*Gpr84*	G protein-coupled receptor 84	5.00	3.65	2.51	2.13	1.64
NM_015811.1	*Rgs1*	Regulator of G-protein signaling 1	2.20	1.15	1.20	-1.07	-1.04
XM_140740.3	*LOC240672*	Similar to MAP-kinase phosphatase (cpg21)	9.96	1.73	1.22	1.02	1.13
**Immunity and defense (17)**							
NM_013652.1	*Ccl4*	Chemokine (C-C motif) ligand 4	4.06	1.36	1.11	1.16	1.36
NM_013693	*Tnf*	Tumor necrosis factor	4.43	1.86	1.67	1.77	1.59
NM_011905.2	*Tlr2*	Toll-like receptor 2	2.19	2.28	1.51	1.30	1.13
NM_007642.2	*Cd28*	CD28 antigen	-1.48	-2.09	-1.82	-1.38	-1.38
M_009856.1	*Cd83*	CD83 antigen	4.06	2.12	-1.24	-1.23	1.04
NM_019948.1	*Clecsf9*	C-type (calcium dependent, carbohydrate recognition domain) lectin, superfamily member 9	3.97	4.17	2.36	1.83	1.71
**Protein metabolism and modification (13)**							
NM_009397.2	*Tnfaip3*	Tumor necrosis factor, alpha-induced protein 3	2.74	1.52	1.11	1.08	1.11
NM_008873.2	*Plau*	Plasminogen activator, urokinase	2.09	1.38	1.32	1.27	1.19
NM_011113.2	*Plaur*	Urokinase plasminogen activator receptor	3.42	2.00	1.22	1.24	1.39
NM_011018.1	*Sqstm1*	Sequestosome 1	2.01	2.17	1.52	1.20	1.03
NM_022331.1	*Herpud1*	Homocysteine-inducible, endoplasmic reticulum stress-inducible, ubiquitin-like domain member 1	2.06	1.07	-1.26	1.16	1.25
**Developmental processes (11)**							
NM_010700	*Ldlr*	Low density lipoprotein receptor	1.34	-2.39	-1.58	-1.11	-1.02
NM_010118.1	*Egr2*	Early growth response 2	6.77	1.95	1.50	1.50	1.50
**Nucleoside, nucleotide, and nucleic acid metabolism (10)**							
NM_007913.2	*Egr1*	Early growth response 1	6.32	-1.32	-1.25	1.11	1.32
NM_018781	*Egr3*	Early growth response 3	6.55	-1.06	-1.28	-1.1	1.08
NM_010657	*Hivep3*	Human immunodeficiency virus type I enhancer binding protein 3	2.28	2.28	1.20	-1.27	-1.22
NM_030612	*Nfkbiz*	Nuclear factor of kappa light polypeptide gene enhancer in B-cells inhibitor, zeta	11.11	2.08	1.69	1.72	2.02
NM_010755.2	*Maff*	V-maf musculoaponeurotic fibrosarcoma oncogene family, protein F (avian)	2.2	1.16	1.12	1.13	1.10
NM_010235.1	*Fosl1*	Fos-like antigen 1	2.3	-1.03	-1.05	-1.03	-1.15
NM_008037.3	*Fosl2*	Fos-like antigen 2	2.56	1.03	-1.03	-1.11	-1.06
**Cell cycle, oncogenesis, cell proliferation and differentiation (13)**							
NM_007669.2	*Cdkn1a*	Cyclin-dependent kinase inhibitor 1A (P21)	2.14	1.15	1.01	1.05	1.02
NM_152804.1	*Plk2*	Polo-like kinase 2	5.77	1.39	-1.03	1.08	1.28
NM_013807.1	*Plk3*	Polo-like kinase 3	2.54	1.25	1.14	1.26	1.27
NM_008416.1	*JunB*	Jun-B oncogene	5.42	1.61	1.21	1.21	1.00
NM_011057.2	*Pdgfb*	Platelet derived growth factor, B polypeptide	4.94	2.45	1.25	1.02	-1.03
NM_018820.3	*Sertad1*	SERTA domain containing 1 (Sertad1)	3.00	1.25	1.06	1.12	1.16
**Cell structure and motility (7)**							
AK031171	*Msn*	Moesin	2.35	1.09	1.13	1.29	1.22
NM_007484.1	*RhoC*	Ras homolog gene family, member C	1.61	2.35	2.02	1.81	1.36
NM_026131	*Pdlim7*	PDZ and LIM domain 7	2.51	3.28	2.38	1.50	1.24
**Other metabolism (8)**							
NM_009834.1	*Ccrn4l*	CCR4 carbon catabolite repression 4-like (*S. cerevisiae*)	2.32	1.54	1.03	1.03	1.01
**Transport (3)**							
NM_015747.1	*Slc20a1*	Solute carrier family 20, member 1	2.62	1.09	-1.14	1.03	1.04
**Lipid, fatty acid and steroid metabolism (3)**							

NM_026644.1	*Agpat4*	1-acylglycerol-3-phosphate O-acyltransferase 1 (lysophosphatidic acid acyltransferase, delta)	2.25	2.21	1.17	-1.06	1.02
NM_134469	*Fdps*	Farnesyl diphosphate synthetase	-1.91	-2.61	-1.62	-1.25	-1.08
**Neuronal activities (2)**							
NM_010119.3	*Ehd1*	EH-domain containing 1	2.72	2.62	1.42	1.23	1.07
NM_007498.2	*Atf3*	Activating transcription factor 3	2.12	1.59	-1.08	-1.16	-1.02
**Cell adhesion (2)**							
NM_010493.2	*Icam1*	Intercellular adhesion molecule	3.05	1.43	1.28	1.22	1.08
NM_027871.1	*Arhgef3*	Rho guanine nucleotide exchange factor (GEF) 3	3.01	2.24	1.51	1.35	1.24
**Muscle contraction (1)**							
NM_011619.1	*Tnnt2*	Troponin T2, cardiac (Tnnt2)	1.84	2.26	2.75	2.46	2.05
**Others and Biological process unclassified (25)**							
NM_021327.1	*Tnip1*	TNFAIP3 interacting protein 1	2.63	2.45	1.8	1.34	1.14
XM_127883	*Irg1*	Immunoresponsive gene 1	5.71	6.77	4.54	3.06	2.33
NM_009672.2	*Anp32a*	Acidic (leucine-rich) nuclear phosphoprotein 32 family, member A	1.01	1.22	1.42	1.30	2.58
AK083478	*Slc11a2*	Solute carrier family 11 (proton-coupled divalent metal ion transporters), member 2	2.05	3.25	2.08	1.77	1.81
NM_009344.1	*Phlda1*	Pleckstrin homology-like domain, family A, member 1	7.53	1.73	1.20	1.14	1.23

Table B. Genes altered in macrophages stimulated with BoNT/A (5 nM) for 2, 4, 6, 8, and 10 h

GenBank ID	Gene symbol	Gene or protein	Fold change (according to treatment time)				
			2 h	4 h	6 h	8 h	10 h
**Signal transduction (103)**							
NM_007707.2	*Socs3*	Suppressor of cytokine signaling 3	12.86	9.73	6.17	5.23	4.44
NM_009421.2	*Traf1*	Tnf receptor-associated factor 1	11.51	4.85	2.85	2.03	1.96
NM_170704.1	*Tnfrst5*	Tumor necrosis factor receptor superfamily, member 5	6.84	10.81	11.12	7.84	4.78
NM_007987.1	*Tnfrsf6*	Tumor necrosis factor receptor superfamily, member 6	3.67	7.02	5.59	3.43	2.11
NM_010090.2	*Dusp2*	Dual specificity phosphatase 2	4.15	2.28	1.79	1.61	1.60
NM_176933	*Dusp4*	Dual specificity phosphatase 4	4.60	1.64	1.55	1.66	1.84
NM_030720.1	*Gpr84*	G protein-coupled receptor 84	8.82	8.12	5.85	4.55	3.07
NM_172161.2	*Irak2*	Interleukin-1 receptor-associated kinase 2	2.74	2.48	2.31	1.61	1.32
NM_015811.1	*Rgs1*	Regulator of G-protein signaling 1	3.01	1.67	1.66	1.72	1.85
XM_140740.3	*LOC240672*	Similar to MAP-kinase phosphatase (cpg21)	30.26	5.40	4.09	4.51	4.37
NM_011756.3	*Zfp36*	Zinc finger protein 36	3.29	2.03	2.09	1.75	1.66
**Immunity and defense (62)**							
NM_011337.1	*Ccl3*	Chemokine (C-C motif) ligand 3	3.14	3.34	2.98	2.96	3.05
NM_013652.1	*Ccl4*	Chemokine (C-C motif) ligand 4	9.66	5.87	5.28	4.61	5.28
NM_013653.1	*Ccl5*	Chemokine (C-C motif) ligand 5	2.60	5.50	6.09	4.92	5.25
NM_011338	*Ccl9*	Chemokine (C-C motif) ligand 9	1.92	2.45	2.54	2.29	1.89
NM_009140	*Cxcl2*	Chemokine (C-X-C motif) ligand 2	11.71	3.10	3.45	4.61	5.57
NM_017466.3	*Ccrl2*	Chemokine (C-C motif) receptor-like 2	6.77	2.72	2.44	1.36	1.19
NM_013693	*Tnf*	Tumor necrosis factor	8.11	5.80	6.56	5.55	4.96
NM_009396.1	*Tnfaip2*	Tumor necrosis factor, alpha-induced protein 2	4.53	3.14	2.03	1.89	2.00
NM_011905.2	*Tlr2*	Toll-like receptor 2	3.43	3.23	1.94	1.54	1.18
NM_007642.2	*Cd28*	CD28 antigen	-2.48	-4.00	-2.30	-2.41	-1.98
XM_132882.1	*Cd69*	CD69 antigen	5.79	4.17	3.18	1.97	1.69
NM_009856.1	*Cd83*	CD83 antigen	9.30	3.07	1.05	-1.34	-1.34
NM_019948.1	*Clecsf9*	C-type (calcium dependent, carbohydrate recognition domain) lectin, superfamily member 9	9.93	9.46	7.41	6.17	4.53
NM_008361	*Il1b*	Interleukin 1 beta	16.52	6.79	4.24	3.31	2.70
NM_010187.1	*Fcgr2b*	Fc receptor, IgG, low affinity IIb	1.63	2.53	2.91	2.93	3.17
NM_008518.1	*Ltb*	Lymphotoxin B	2.17	3.31	3.36	3.03	2.33
NM_133662.1	*Ier3*	Immediate early response 3	2.66	2.29	2.65	2.55	2.79
NM_008332.2	*Iift2*	Interferon-induced protein with tetratricopeptide repeats 2	-1.04	5.74	10.46	5.33	2.78
NM_010501.1	*Iift3*	Interferon-induced protein with tetratricopeptide repeats 3	-1.40	4.59	8.07	3.59	2.36
AK037454	*Fyb*	FYN binding protein	3.73	2.69	2.10	2.25	2.14
NM_145209.2	*Oasl1*	2-5 oligoadenylate synthetase-like 1	2.05	3.96	5.36	3.08	1.89
NM_013532.1	*Lilrb4*	Leukocyte immunoglobulin-like receptor B4	3.20	3.24	3.09	3.04	3.85
NM_008147.1	*Gp49a*	Glycoprotein 49 A	2.94	2.60	2.83	3.01	3.78
NM_018734.2	*Gbp4*	Guanylate-binding protein-4	1.54	3.58	5.48	3.94	2.62
NM_011315	*Saa3*	Serum amyloid A 3	3.61	8.29	9.86	9.13	9.39
**Protein metabolism and modification (28)**							
NM_009397.2	*Tnfaip3*	Tumor necrosis factor, alpha-induced protein 3	6.86	3.12	2.32	1.98	2.41
NM_008873.2	*Plau*	Plasminogen activator, urokinase	2.20	1.36	1.35	1.47	1.51
NM_011113.2	*Plaur*	Urokinase plasminogen activator receptor	7.29	4.85	4.30	4.63	4.41
NM_011018.1	*Sqstm1*	Sequestosome 1	3.60	3.65	2.89	2.34	2.27
NM_022331.1	*Herpud1*	Homocysteine-inducible, endoplasmic reticulum stress-inducible, ubiquitin-like domain member 1	4.37	1.90	1.77	2.49	2.37
NM_011414.1	*Slpi*	Secretory leukocyte protease inhibitor	1.59	2.23	3.31	3.90	4.42
NM_013599.2	*Mmp9*	Matrix metalloproteinase 9	2.87	3.91	4.27	4.32	3.71
NM_145933.2	*St6gal1*	Beta galactoside alpha 2,6 sialyltransferase 1	-1.53	-2.94	-3.14	-3.29	-3.65
NM_011909.1	*Usp18*	Ubiquitin specific protease 18	-1.64	1.86	4.46	3.41	2.28
NM_015783.1	*G1p2*	Interferon, alpha-inducible protein	1.92	5.10	8.56	6.23	4.20
NM_030701.1	*Gpr109b*	G protein-coupled receptor 109B	5.09	5.72	5.34	5.43	4.95
**Developmental processes (22)**							

NM_010128.3	*Emp1*	Epithelial membrane protein 1	-1.4	-1.21	-2.1	-2.85	-3.37
NM_011408.1	*Slfn2*	Schlafen 2	3.88	4.03	4.87	3.5	2.82
NM_010128.3	*Emp1*	Epithelial membrane protein 1	-1.4	-1.21	-2.1	-2.85	-3.37
**Nucleoside, nucleotide, and nucleic acid metabolism (33)**							
NM_007913.2	*Egr1*	Early growth response 1	3.12	-1.51	1.66	1.42	1.51
NM_018781	*Egr3*	Early growth response 3	10.31	1.15	1.41	1.50	1.25
NM_010657	*Hivep3*	Human immunodeficiency virus type I enhancer binding protein 3	4.97	4.50	2.30	1.47	1.24
NM_015786	*Hist1h1c*	Histone 1, H1c	3.49	2.52	1.45	-1.28	-2.12
NM_030612	*Nfkbiz*	Nuclear factor of kappa light polypeptide gene enhancer in B-cells inhibitor, zeta	13.63	6.02	5.34	4.84	4.63
NM_010755.2	*Maff*	V-maf musculoaponeurotic fibrosarcoma oncogene family, protein F (avian)	2.20	1.16	1.12	1.13	1.10
NM_010235.1	*Fosl1*	Fos-like antigen 1	3.63	1.96	2.14	2.71	2.79
NM_008037.3	*Fosl2*	Fos-like antigen 2	3.04	1.24	1.21	1.43	1.40
NM_007528.1	*Bcl6b*	B-cell CLL/lymphoma 6, member B	4.14	2.41	2.53	2.56	2.77
NM_007913.2	*Egr1*	Early growth response 1	3.12	-1.51	1.66	1.42	1.51
**Cell cycle, oncogenesis, cell proliferation and differentiation (45)**							
NM_007669.2	*Cdkn1a*	Cyclin-dependent kinase inhibitor 1A (P21)	4.08	2.63	2.85	2.78	3.74
NM_152804.1	*Plk2*	Polo-like kinase 2	9.34	2.23	2.65	4.51	5.66
NM_013807.1	*Plk3*	Polo-like kinase 3	2.89	1.68	2.28	2.49	2.45
NM_008416.1	*JunB*	Jun-B oncogene	7.66	2.61	2.28	1.89	1.68
NM_011057.2	*Pdgfb*	Platelet derived growth factor, B polypeptide	14.59	6.60	2.54	1.97	1.66
NM_018820.3	*Sertad1*	SERTA domain containing 1 (Sertad1)	2.82	1.76	1.71	1.72	1.97
NM_009971.1	*Csf3*	Colony stimulating factor 3 (granulocyte)	72.38	33.68	59.10	59.89	97.82
NM_008655.1	*Gadd45b*	Growth arrest and DNA-damage-inducible 45 alpha	6.45	2.17	1.85	1.75	1.66
NM_009046.2	*Relb*	Avian reticuloendotheliosis viral (v-rel) oncogene related B	3.71	1.85	1.19	1.15	-1.06
NM_008491.1	*Lcn2*	Lipocalin 2	1.06	1.86	2.73	2.96	3.48
NM_018807.3	*Plagl2*	Pleiomorphic adenoma gene-like 2	2.34	2.24	2.09	2.15	1.94
NM_009506.1	*Vegfc*	Vascular endothelial growth factor C	2.40	4.55	4.66	3.33	2.72
NM_008842.2	*Pim1*	Proviral integration site 1	4.59	1.68	1.35	1.14	1.11
NM_033563	*Klf7*	Kruppel-like factor 7 (ubiquitous)	2.83	1.64	1.52	1.47	1.34
NM_007669.2	*Cdkn1a*	Cyclin-dependent kinase inhibitor 1A (P21)	4.08	2.63	2.85	2.78	3.74
**Cell structure and motility (14)**							
AK031171	*Msn*	Moesin	2.74	1.58	1.76	1.66	1.51
NM_007484.1	*RhoC*	Ras homolog gene family, member C	2.28	2.89	2.90	2.78	2.43
NM_026131	*Pdlim7*	PDZ and LIM domain 7	3.78	3.58	2.41	1.92	1.72
NM_026772.1	*Cdc42ep2*	CDC42 effector protein (Rho GTPase binding) 2	5.33	2.59	2.86	2.44	1.99
**Apoptosis (24)**							
NM_007534	*Bcl2a1b*	B-cell leukemia/lymphoma 2 related protein A1b	2.61	3.24	3.04	2.41	2.17
NM_007536	*Bcl2a1d*	B-cell leukemia/lymphoma 2 related protein A1d	2.85	3.52	3.09	2.44	2.15
NM_178045.3	*Rassf4*	Ras association (RalGDS/AF-6) domain family 4	3.67	5.03	3.30	2.39	1.64
**Blood circulation and gas exchange (1)**							
NM_010104.2	*Edn1*	Endothelin 1	3.68	3.21	2.03	1.78	1.64
**Nitrogen metabolism (1)**							
NM_010927.1	*INOS*	Nitric oxide synthase 2, inducible, macrophage	4.36	5.72	5.72	4.62	4.26
**Other metabolism (15)**							
NM_009834.1	*Ccrn4l*	CCR4 carbon catabolite repression 4-like (*S. cerevisiae*)	4.09	1.95	1.17	1.10	1.29
NM_010442.1	*Hmox1*	Heme oxygenase (decycling) 1	1.43	1.29	2.88	4.51	4.84
NM_053108	*Glrx1*	Glutaredoxin 1 (thioltransferase)	2.95	3.89	3.54	3.68	4.90
NM_009183.1	*Siat8d*	Sialyltransferase 8 (alpha-2, 8-sialyltransferase) D	-2.85	-2.06	-1.65	-1.78	-1.55
**Transport (16)**							
NM_015747.1	*Slc20a1*	Solute carrier family 20, member 1	3.45	1.27	1.76	2.06	2.11
NM_080853	*Slc17a6*	solute carrier family 17 (sodium-dependent inorganic phosphate cotransporter), member 6	1.22	1.58	1.95	2.43	3.31
NM_025286.1	*Slc31a2*	Solute carrier family 31, member 2	3.09	3.27	3.05	2.77	2.71
NM_011990.1	*Slc7a11*	Solute carrier family 7 (cationic amino acid transporter, y+ system), member 11	1.53	2.13	2.03	2.29	3.44
**Lipid, fatty acid and steroid metabolism (10)**							
NM_026644.1	*Agpat4*	1-acylglycerol-3-phosphate O-acyltransferase 1 (lysophosphatidic acid acyltransferase, delta)	5.91	4.64	1.94	1.70	1.94
NM_134469	*Fdps*	Farnesyl diphosphate synthetase	-1.91	-2.61	-1.62	-1.25	-1.08

NM_138650	*Dgkg*	Diacylglycerol kinase, gamma	-1.14	-3.48	-1.85	-1.61	-1.53
**Neuronal activities (4)**							
NM_010119.3	*Ehd1*	EH-domain containing 1	5.57	4.44	2.72	2.29	2.16
NM_007498.2	*Atf3*	Activating transcription factor 3	3.10	2.36	2.11	2.67	2.35
NM_001039537.1	*Lif*	Leukemia inhibitory factor (Lif), transcript variant 2	16.86	3.77	1.70	1.51	1.29
NM_007413.2	*Adora2b*	Adenosine A2b receptor	3.94	2.09	1.61	1.55	1.54
**Intracellular protein traffic (10)**							
XM_203312.2	*Stx11*	Syntaxin 11	4.48	2.01	1.40	1.47	1.72
**Cell adhesion (3)**							
NM_010493.2	*Icam1*	Intercellular adhesion molecule	4.29	2.55	1.87	1.38	1.28
NM_027871.1	*Arhgef3*	Rho guanine nucleotide exchange factor (GEF) 3	7.46	6.63	4.20	2.95	2.01
NM_010577.2	*Itga5*	Integrin alpha 5	5.11	3.02	1.70	1.56	1.39
**Muscle contraction (2)**							
NM_011619.1	*Tnnt2*	Troponin T2, cardiac	1.99	2.78	3.34	3.16	2.83
**Others and Biological process unclassified (84)**							
NM_021327.1	*Tnip1*	TNFAIP3 interacting protein 1	4.38	3.47	2.84	2.46	2.15
XM_127883.4	*Irg1*	Immunoresponsive gene 1	62.32	85.25	78.25	58.61	34.67
AK083478	*Slc11a2*	Solute carrier family 11 (proton-coupled divalent metal ion transporters), member 2	5.22	5.06	3.73	3.54	3.64
NM_016917.1	*Slc40a1*	Solute carrier family 40 (iron-regulated transporter), member 1	-1.62	-2.29	1.07	2.64	3.19
NM_009344.1	*Phlda1*	Pleckstrin homology-like domain, family A, member 1	22.79	5.33	6.20	6.31	6.55
NM_020557.3	*Tyki*	Thymidylate kinase family LPS-inducible member	-1.52	3.25	4.75	2.53	2.00
NM_008326.1	*Ifi1*	Interferon inducible protein 1	-1.92	1.66	3.32	2.23	1.80
M64404	*Il1ra*	IL-1 receptor antagonist	4.23	5.03	6.09	7.32	6.25
NM_019549	*Plek*	Pleckstrin	2.68	2.40	2.13	2.56	2.53
XM_125538.4	*Sesn1*	Sestrin 1	-4.70	-2.05	-1.71	-2.00	-2.03
XM_137493	*Osm*	Oncostatin M	4.65	2.08	2.25	2.77	2.87
AK045226	*Cd44*	CD44 antigen	2.35	2.56	2.06	1.81	1.39
XM_207780.2	*Cd101*	Immunoglobulin superfamily, member 2	-1.30	-2.01	-2.54	-2.88	-3.44
AK052726	*Nfkb1*	Nuclear factor of kappa light chain gene enhancer in B-cells 1, p105	4.16	1.98	1.68	1.44	1.29
NM_019738.1	*Nupr1*	Nuclear protein 1	3.51	2.70	1.65	1.18	1.19
AK011999	*Pde4b*	Phosphodiesterase 4B, cAMP specific	4.03	2.11	1.57	1.38	1.27
NM_023324.1	*Peli1*	Pellino 1	3.12	2.80	1.94	1.44	1.36
